# Optimizing antithrombotic therapy following mitral valve repair: a comprehensive network meta-analysis

**DOI:** 10.1186/s12872-025-04974-4

**Published:** 2025-08-23

**Authors:** Mohamed Ibrahim Gbreel, Mohamed Hamouda Elkasaby, Marwa Hassan, Marc Ulrich Becher, Mahmoud Balata

**Affiliations:** 1 Faculty of Medicine and Surgery, October 6 University, Giza, Egypt; 2 Department of Cardiology, Epyptian Railway Medical Centre (ERMC), Cairo, Egypt; 3 Faculty of Medicine and Surgery, Al-Azhar University, Cairo, Egypt; 4 Department of Immunology, Theodor Bilharz Research Institute, Giza, Egypt; 5 Department of Cardiology, Städtisches Klinikum Solingen, Solingen, Germany; 6 Department of cardiology, University hospital Rostock, Rostock, Germany

**Keywords:** ASA, Clopidogrel, Anticoagulant, Mitral valve repair, Mitral regurgitation, Network meta-analysis

## Abstract

**Background:**

Mitral regurgitation (MR) presents either as primary or secondary, with options for surgical or transcatheter repair. Thromboembolic risks following surgery are significant despite the use of antithrombotic medications, and guidelines for postoperative anticoagulation therapy lack consistency. This systematic review aims to compare antithrombotic medications after mitral valve repair (MVR). In this study, we intend to compare antithrombotic medications after MVR.

**Materials and methods:**

The study followed the Cochrane handbook and PRISMA guidelines. We systematically searched databases (PubMed, Scopus, Ovid, Cochrane, Web of Science) until June 2024 for TMVR studies using specific criteria. Quality assessment utilized the Newcastle-Ottawa scale. Data extraction encompassed study characteristics and outcomes. Primary outcomes included thromboembolic events and bleeding within six months. Statistical analysis employed R software to assess heterogeneity and publication bias.

**Results:**

From the 121 articles screened, 12 were included in the study. These cohort studies, involving 20,644 participants, spanned from 2008 to 2022. While most studies were of good to high quality, some exhibited lower quality. Analysis favored oral anticoagulants (OAC) over single antiplatelet therapy (SAPT) for reducing bleeding risk (RR = 0.31, 95% CI: [0.11–0.87], *P* < 0.05), with moderate heterogeneity. Thromboembolic events did not significantly differ among interventions. Transient ischemic attacks and stroke outcomes were similar between SAPT and vitamin K antagonists (VKA). Six-month mortality rates were comparable between SAPT and VKA, with notable heterogeneity and higher mortality with SAPT in one study. Qualitative synthesis highlighted procedural success rates and bleeding complications across different interventions in transcatheter mitral valve repair studies.

**Conclusion:**

OACs showed a lower risk of bleeding compared to antiplatelet therapies, while VKAs and OAC + SAPT may reduce thromboembolic events. No significant differences were found in stroke, TIA, or short-term mortality. These findings support individualized therapy and highlight the need for further randomized trials.

**Supplementary Information:**

The online version contains supplementary material available at 10.1186/s12872-025-04974-4.

## Introduction

Mitral regurgitation (MR) is one of the most prevalent valvular heart diseases, affecting more than twenty-four million people worldwide, and is associated with high mortality and morbidity rates [1]. Pathological changes in the mitral valve cause primary MR. In the Western world, degenerative changes are the most common cause, while in low-income countries, rheumatic heart disease is the leading cause. [2–4]. On the other hand, secondary MR is the result of abnormal changes in the left ventricle, where the MV is normal. It can also be caused by atrial fibrillation (AF) or left atrial enlargement [5].

Current international guidelines offer varying recommendations regarding the choice and duration of antithrombotic therapy post-MVR. The 2020 ACC/AHA guidelines suggest oral anticoagulation for at least 3 months after mitral valve repair (MVR) in patients without other indications for long-term anticoagulation, with a preference for warfarin (Class IIa recommendation) by Otto CM et al., 2021. Conversely, the 2017 ESC/EACTS guidelines are less specific, recommending individualized decision-making based on patient risk profiles and procedural factors, without a strong preference for anticoagulation versus antiplatelet therapy. These discrepancies highlight the ongoing uncertainty surrounding optimal postoperative antithrombotic strategies.”

The conjoint guidelines of the European Heart Association and the European Association for Cardio-Thoracic Surgery (ESC-EACTS) [6] and the American Guidelines of the American College of Cardiology and the American Heart Association (ACC-AHA) [7] recommend surgical mitral valve repair (SMVR) as the gold standard treatment for symptomatic MR for operable patients, while the transcatheter MV repair (PMVR) is typically recommended for those who deemed inoperable or high surgical risk. Thromboembolic (TE) events following SMVR vary from 0.4 to 1.6% per year and increase to 2.5% during the first month after the surgery, even with routine antithrombotic (AT) medications [8].

After MVR, consistent guidelines on anticoagulation therapy are lacking, leading to varying clinical practices. Interestingly, In A large cohort of patients in North America who underwent MVR between 2008 and 2010, a real-world practice patterns, as described by Suri et al. [9], demonstrate that anticoagulation decisions are often driven by institutional or physician preferences rather than patient-specific characteristics, further emphasizing the need for more robust comparative evidence [9].

The recommendations provided by international guidelines for postoperative antithrombotic management have been controversial as well [9]. In this systematic review and network meta-analysis, we aim to compare different AT medications after MVR in a network meta-analysis.

## Materials and methods

We followed the approaches for conducting the current study based on the Cochrane handbook of systematic reviews on interventions [10]. During the drafting of our manuscript, we strictly followed the recommended reporting items for the Preferred Reporting Items for Systematic Reviews and Meta-Analyses (PRISMA) statement guidelines [11].

### Search strategy

The following electronic databases were systematically searched: PubMed, Web of Science (WOS), Scopus, Ovid, and Cochrane till 27 June 2024. We used the following search strategy for searching on prespecified databases: (Acetylsalicylic OR Acylpyrin OR Aloxiprimum OR Colfarit OR Dispril OR Easprin OR Ecotrin OR Endosprin OR Magnecyl OR Micristin OR Polopirin OR Polopiryna OR Solprin OR Solupsan OR Zorprin OR Acetysal OR lysine OR aspirin OR aspiryl-polylysine OR Solusprin OR Aspisol OR Flectadol OR Solusprin OR Venopirin OR Aspegic OR Clopidogrel OR Iscover OR PCR OR PlaviX OR *Clogrel* OR SC 25989 C OR SR 25989) AND (Apo-Warfarin OR Aldocumar OR Gen-Warfarin OR Warfant OR Coumadin OR Marevan OR Warfarin OR Coumadine OR Tedicumar OR Pradaxa OR Dabigatran OR rivaroxaban OR Xarelto OR Eliquis OR apixaban OR edoxaban OR Savaysa OR Lixiana OR Roteas OR *Betrixaban* OR Bevyxxa) AND (Mitral OR Bicuspid OR TMVR OR Repair). All the included studies’ references were screened to avoid missing any studies and guarantee high-quality screening.

### Eligibility criteria

We included studies that enrolled adult patients who underwent MVR either through a surgical approach (SMVR) or transcatheter techniques (TMVR). The primary focus was on TMVR. The data from surgical cohorts were considered mainly in secondary and comparative analyses when relevant. Eligible studies had to evaluate antithrombotic interventions, specifically acetylsalicylic acid (ASA), clopidogrel, vitamin K antagonists (VKA), or other oral anticoagulants (OACs), alone or in combination. We included both randomized controlled trials (RCTs) and prospective or retrospective cohort studies that reported at least one of the predefined outcomes of interest. We excluded studies based on the following criteria: non-human studies, case reports, case series, conference abstracts, editorials, reviews, studies lacking original patient data, and non-English publications. Duplicate publications and studies with overlapping data were assessed carefully.

### Screening and study selection

Using EndNote software [12]We gathered various records from multiple databases and eliminated duplicates. The retrieved references underwent screening to assess their relevance. This process was conducted in two steps: first, title and abstract screening, followed by full-text screening for final eligibility. The interventions included in the final analysis after screening were dual antiplatelet therapy (DAPT), Warfarin (VKA), Aspirin/single antiplatelet therapy (SAPT), oral anticoagulant (OAC), and no antiplatelet therapy (No APT). At least two independent authors performed each step, and their findings were compared; group discussions resolved any disagreements.

### Quality assessment

An adapted version of the Newcastle-Ottawa scale (NOS) quality assessment [13] for cohort studies was used for quality assessment of each included study. The scale encompasses the following domains: Sample selection criteria, Comparability and Exposure. It adopts a star-awarding system for specific methodological sections, which permits calculating an overall quality score for each study. The scale gives a maximum score of 10 points, where the studies were categorized according to their score as follows: high risk of bias (0–3 points), moderate risk of bias (4–6 points), or low risk of bias (≥ 7 points). Any disagreements during the quality assessment were resolved through mutual consensus and/or by consulting the third senior author.

### Data extraction

Two independent authors extracted the following data from the included studies. Extracting summary of included studies included the design, setting, sample size, follow-up duration, population definition, outcomes, and baseline characteristics of the patients in the included studies.

### Endpoints

The primary outcomes of our study included thromboembolic events, bleeding events, transient ischemic attacks (TIAs), stroke, and mortality events within six months.

### Statistical analysis

We used R version 4.2.2 (2022-10-31) [14], and R Studio version 2022.07.2 (2009–2022) RStudio, Inc.) [15]. Dichotomous data were analyzed as risk ratio (RR) and 95% confidence interval (CI) and continuous data as mean difference (MD) and 95% CI. Statistical heterogeneity among the studies was assessed by visual inspection of the forest plot, besides using I-squared (I^2^) and chi-squared (Chi^2^) statistics. I^2^ values of 50% were indicative of high heterogeneity. A random-effects model was applied when there was a significant variation in the data. Other than that, the fixed-effect model was applied. Egger test was used to assess the publication bias [16].

## Results

### Literature search

**Fig. 1:**
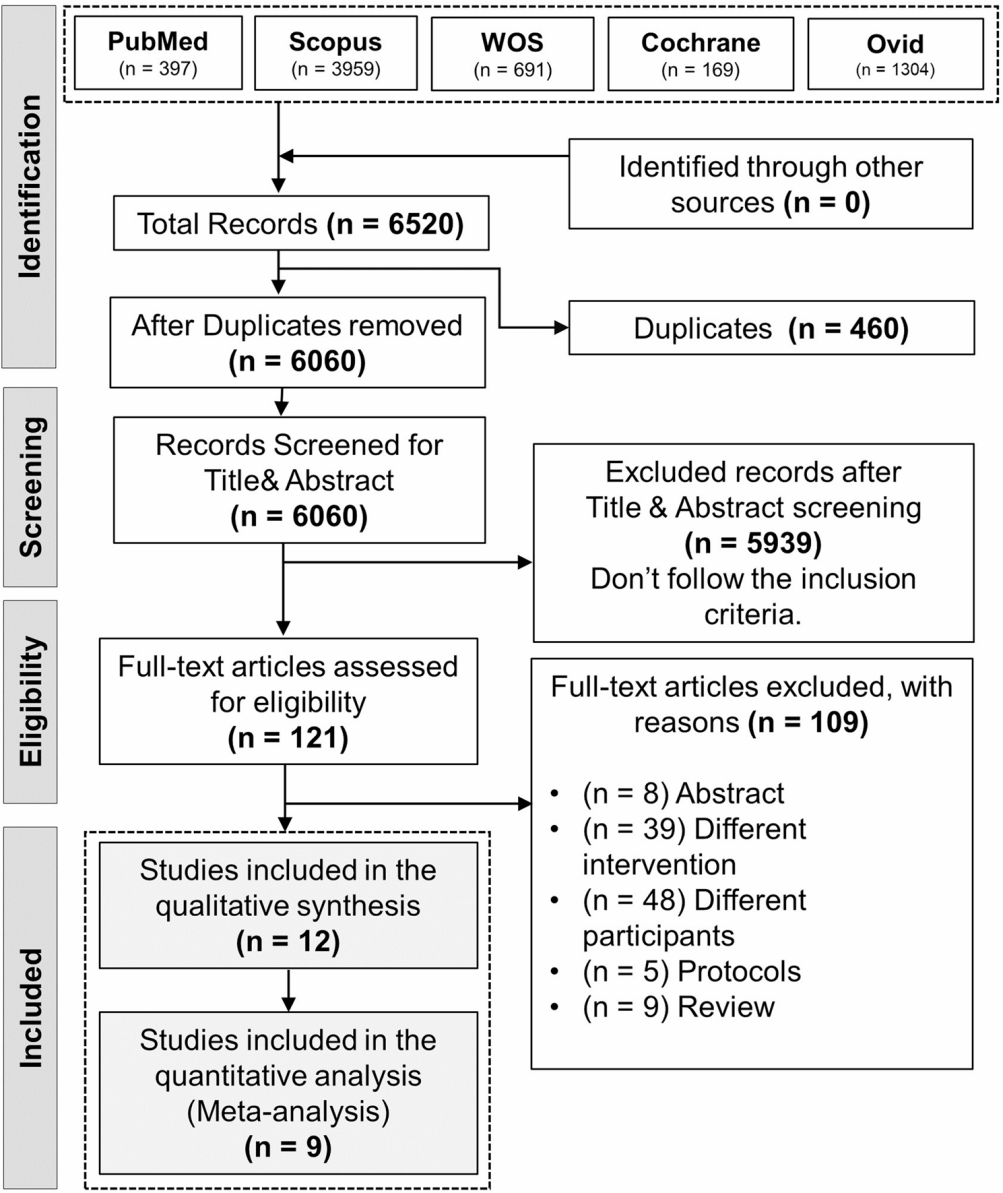
PRISMA Flow Diagram, showing the systematic review process, including initial identification, screening, eligibility assessment, and final study inclusion

The computerized search and reference lists of the databases yielded 6520 articles. After duplicates were removed, 6060 articles were screened. Following the primary screening, 121 articles were included. The full texts of these papers were processed to secondary screening, with 12 articles [17–28] included in the study and nine [17–21, 23–27] included in the analysis. (Fig. 1: PRISMA flow diagram.

### Characteristics of the included studies

A total of 20,644 were included in the study and 19,294 were included in the screening. All these studies were cohort studies either prospective or retrospective done between 2008 and 2022. The follow-up durations ranged between 45 days to 10 years. For in-depth insight into the participants, Table 1 and 2 presents detailed characteristics.

**Table 1:** Summary of the included studies

Study ID	Year	Setting	Design	Sample Size	Follow-up duration	Type of repair	Population definition	Primary outcome measures
(Benito-González, et al.) [28]	2018	Spain	Cohort	80	523.5 days	Percutaneous	Patients underwent PMVR.	Admission for HF and all-cause mortality.
(Körber, et al.) et al. [35]	2018	Germany	Cohort	347	1 Year	Percutaneous	Patients who underwent PMVR.	Occurrence of bleeding.
Hohmann et al. [27]	2022	Germany	Cohort	1342	6 months	Percutaneous	Patients (≥ 18 years of age) who received MitraClip for the first time.	(1) MACE defined as a combined endpoint of cardiovascular mortality, MI, and ischemic stroke, and (2) All-cause mortality (3) intracranial bleeding, (4) major extracranial bleeding and GIT bleeding.
Meurin et al. [25]	2008	Multi-centric	Cohort	350	45 days	N/A	Patients underwent MVR	Occurrence of thromboembolic events within 6 weeks of the procedure.
Paparella et al. [33]	2016	Multi-centric	Cohort	1882	6 months	Percutaneous	Patients aged more than or equal to 18 years at time of surgery underwent isolated MVR with mitral ring implantation to treat MR, and in sinus rhythm at hospital discharge.	Primary efficacy outcome was the incidence of arterial thromboembolic events within 6 months and primary safety outcome was the incidence of major bleeding within 6 months.
Seeger et al. [22]	2019	Germany	Cohort	254	10 years	N/A	Patients underwent TMVR.	All-cause mortality, all stroke, and rehospitalization for congestive heart failure or myocardial infarction.
Suri et al. [9]	2013	North America	Cohort	13,082	NA	N/A	Patients who underwent primary mitral valve repair. Those patients having other major concomitant operations or with an indication/contraindication to warfarin were excluded.	Complications (AF, Permanent stroke, Reoperation for bleeding/tamponade), length of hospital stay, use of blood products, medications at discharge (Aspirin, or Beta-blockers)
Waechter et al. [19]	2022	Germany	Cohort	609	419 days	TEER	Patients undergoing TEER with the MitraClip.	All-cause in-hospital death and all-cause mortality during follow-up.
Van der Wall et al. [31]	2018	Netherlands	Cohort	469	3 months	N/A	Patients with mitral valve repair	The combined incidence of thromboembolic and bleeding complications.
Noohi et al. [24]	2020	Iran	Cohort	297	1 year	NA	Patients undergoing mitral valve repair.	Safety and efficacy of rivaroxaban.
Valeur et al. [20]	2016	Denmark	Cohort	2188	4.9 years	NA	Patients who underwent mitral valve repair.	Mortality and stroke
Watt et al. [17]	2020	USA	Cohort	1097	10 years	NA	Patients who underwent elective mitral valve repair.	Occurrence of thromboembolic events.
*HF*, Heart failure, *PMVR*, percutaneous edge-to-edge mitral valve repair, *MACE*, Major adverse cardiovascular events, *MI*, myocardial infarction, *MVR*, mitral valve repair, *MR*, mitral regurgitation, *TMVR*, transcatheter mitral valve repair, *AF*, Atrial fibrillation, *TEER*, Transcatheter Edge-to-Edge Repair								

**Table 2:** Baseline characteristics of the included studies

Study ID	Arms	Sample	Age, y mean (SD)	Gender, Male, *n* (%)	BMI, kg/m2 mean(SD)	Comorbidities *n*(%)				Echocardiographic measures			
						AF	DM	HTN	Stroke	Functional MR etiology, *n* (%)	LVEF, % mean(SD)	Transmitral mean gradient, mm Hg mean(SD)	Pulmonary artery systolic pressure, mm Hg mean(SD)
Benito-González et al. 2018 [28]	SAPT, DAPT, VKA, and Dual OAC	80	74.6 (10.1)	52 (65)	26.7 (5.1)	47 (58.8)	23 (28.8)	52 (65)	3 (3.6)	60 (75)	N/A	N/A	46.9 (16.2)
Hohmann et al. 2022 [27]	DAPT	157	74.6 (10.4	103 (65.6)	N/A	35 (22.3)	74 (47.1)	134 (85.4)	N/A	N/A	N/A	N/A	N/A
	Mono OAC	261	77.8 (7.4)	152 (58.2)	N/A	206 (78.9)	117 (44.8)	243 (93.1)	N/A	N/A	N/A	N/A	N/A
	Duo OAC	279	77.0 (8.2)	168 (60.2)	N/A	207 (74.2)	128 (45.9)	253 (90.7)	N/A	N/A	N/A	N/A	N/A
	Triple OAC	37	77.9 (6.8)	25 (67.6)	N/A	23 (62.2)	16 (43.2)	33 (89.2)	N/A	N/A	N/A	N/A	N/A
	No APT	282	75.9 (9.7)	179 (63.5)	N/A	202 (71.6)	116 (41.1)	256 (90.8)	N/A	N/A	N/A	N/A	N/A
Körber et al. 2018 [26]	VKA, OAC, SAPT, and DAPT	347	76 (13.7)	203 (58.5)	25.3 (4.7)	194 (55.9%)	89 (25.6%)	238 (68.6%)	44 (12.7%)	183 (52.7%)	N/A	N/A	N/A
Meurin et al. 2008 [25]	VKA	230	59 (14)	150 (65%)	N/A	53 (23)	N/A	N/A	N/A	N/A	56 (11)	2.8 (1.7)	N/A
	No APT	35	60 (15)	25 (71%)	N/A	0 (0)	N/A	N/A	N/A	N/A	57 (10)	3.2 (2.1)	N/A
Paparella et al. 2016 [33]	SAPT	286	56.3 (17.3)	180 (62.9)	25.4 (4.0)	N/A	16 (5.6)	145 (50.7)	5 (1.7)	N/A	N/A	N/A	N/A
	VKA	858	57.7 (12.9)	539 (62.8)	25.0 (4.8)	N/A	42 (4.9)	404 (47.1)	11 (1.3)	N/A	N/A	N/A	N/A
Seeger et al. 2019 [22]	OAC	136	N/A	N/A	N/A	N/A	N/A	N/A	N/A	N/A	N/A	N/A	N/A
	SAPT	118	N/A	N/A	N/A	N/A	N/A	N/A	N/A	N/A	N/A	N/A	N/A
Suri et al. 2013 [9]	VKA	5,963	59.67 (13.35)	3,578 (60.0)	N/A	N/A	513 (8.6)	3,305 (55.4)	N/A	N/A	59.33 (8.9)	N/A	N/A
	SAPT	7,119	57.33 (12.6)	4,148 (58.3)	N/A	N/A	635 (8.9)	3,933 (55.2)	N/A	N/A	59 (8.16)	N/A	N/A
Waechter et al. 2022 [19]	No APT	11	72.5 (11)	8 (72.7)	N/A	9 (81.8)	3 (27.3)	4 (36.4)	0 (0)	11 (100)	N/A	N/A	N/A
	OAC	146	78.9 (7.5)	86 (58.9)	N/A	135 (92.5)	46 (31.5)	119 (81.5)	14 (9.6)	85 (58.2)	N/A	N/A	N/A
	OAC + SAPT	248	78.4 (8)	157 (63.3)	N/A	234 (94.4)	78 (31.5)	216 (87.1)	31 (12.5)	144 (58.1)	N/A	N/A	N/A
van der Wall et al. 2018 [31]	VKA	325	60 (13)	195 (60)	N/A	N/A	17 (5.4)	149 (47)	7 (2.2)	N/A	N/A	N/A	N/A
	SAPT	144	62 (11)	85 (59)	N/A	N/A	5 (3.5)	74 (51)	8 (5.6)	N/A	N/A	N/A	N/A
Noohi et al. 2020 [36]	OAC	153	49.9 ± 15	60 (39.5%)	N/A	24 (15.7%)	7 (4.9%)	35 (22.9%	N/A	N/A	44.01 ± 10.4	N/A	N/A
	VKA	144	51.9 ± 14.3	51 (35.4%)	N/A	25 (17.4%)	34 (22.2%)	58 (40.3%)	N/A	N/A	46.66 ± 9.2	N/A	N/A
Valeur et al. 2016 [20]	SAPT	1437	Median, 64	960 (67%)	N/A	158 (11%)	175 (12%)	N/A	73 (5%)	N/A	N/A	N/A	N/A
	VKA	751	Median, 63	559 (74%)	N/A	86 (10%)	59 (8%)	N/A	19 (3%)	N/A	N/A	N/A	N/A
Watt et al. 2020 [32]	VKA	775	57 ± 13	442(57.0%)	N/A	N/A	74(9.6%)	347(44.8%)	N/A	N/A	57 ± 12%	N/A	N/A
	SAPT	322	55 ± 15	168(52.2%)	N/A	N/A	38(11.8%)	153(47.5%)	N/A	N/A	57 ± 15%	N/A	N/A
*BMI*, Body mass index, *AF*, Atrial Fibrillation, *HTN*, Hypertension, *DM*, Diabetes mellitus, *MR*, mitral regurgitation, *LVEF*, Left ventricular ejection fraction, *DAPT*, Dual antiplatelet therapy, *SAPT*, Single antiplatelet therapy, *OAC*, oral anticoagulant, *VKA*, Vitamin K antagonist, *APT*, antiplatelet therapy													

### Risk of bias assessment

According to the Newcastle–Ottawa scale, all included studies where good to high quality except Suri et al. 2013 [21], Valeur et al. 2016 [20], and Seeger et al. 2019 [22] were poor quality and Körber et al. 2018 [26] was fair quality. Detailed information about quality assessment is reported in Table 3.

**Table 3:** Riskof Bias Assessment: summarizing the quality assessment results of each study using the Newcastle-Ottawa scale, categorizing them by risk level

Study ID	Hohmann et al. [27]	Wall et al. [31]	Benito-González et al. [28]	Suri et al. [9]	Paparella et al. [23]	Körber et al. [26]	Waechter et al. [29]	Noohi et al. [36]	Valeur et al. [20]	Watt et al. [32]	Seeger et al. [22]	Meurin et al. [25]
Sample selection criteria (****)	****	****	****	****	****	****	****	****	****	****	****	****
1) Representativeness of the exposed cohort (a) Truly representative (one star) (b) Somewhat representative (one star) (c) Selected group (d) No description of the derivation of the cohort	*	*	*	*	*	*	*	*	*	*	*	*
2) Selection of the non-exposed cohort (a) Drawn from the same community as the exposed cohort (one star) (b) Drawn from a different source (c) No description of the derivation of the non-exposed cohort	*			*	*		*	*	*	*	*	
3) Ascertainment of exposure (a) Secure record (e.g., surgical record) (one star) (b) Structured interview (one star) (c) Written self-report (d) No description (e) Other	*	*	*		*		*	*		*		*
4) Demonstration that outcome of interest was not present at start of study (a) Yes (one star) (b) No	*	*	*	*	*	*	*	*	*	*	*	*
Comparability (**)	**	**	**	**	**	**	**	**	**	**	**	**
1) Comparability of cohorts on the basis of the design or analysis controlled for confounders (a) The study controls for age, sex and marital status (one star) (b) Study controls for other factors (list) (one star) (c) Cohorts are not comparable on the basis of the design or analysis controlled for confounders	*	**	**	*	**	**	**	**		*		**
Exposure (***)	***	***	***	***	***	***	***	***	***	***	***	***
1) Assessment of outcome (a) Independent blind assessment (one star) (b) Record linkage (one star) (c) Self report (d) No description (e) Other	*	*		*	*	*	*	*		*		*
2) Was follow-up long enough for outcomes to occur (a) Yes (one star) (b) No	*	*	*		*	*	*	*	*	*	*	*
3) Adequacy of follow-up of cohorts (a) Complete follow up- all subject accounted for (one star) (b) Subjects lost to follow up unlikely to introduce bias- number lost less than or equal to 20% or description of those lost suggested no different from those followed. (one star) (c) Follow up rate less than 80% and no description of those lost (d) No statement		*	*				*		*		*	
Summary quality score	Good	Good	Good	Poor	Good	Fair	Good	Good	Poor	Good	Poor	Good
ID, identification Score: Good quality: 3 or 4 stars in selection domain AND 1 or 2 stars in comparability domain AND 2 or 3 stars in outcome/exposure domain Fair quality: 2 stars in selection domain AND 1 or 2 stars in comparability domain AND 2 or 3 stars in outcome/exposure domain Poor quality: 0 or 1 star in selection domain OR 0 stars in comparability domain OR 0 or 1 stars in outcome/exposure domain												

### Outcomes

#### Bleeding events

**Fig. 2:**
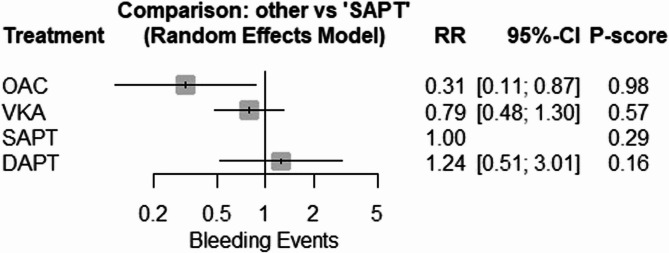
Network Meta-Analysis of Bleeding Events: illustrating the risk ratio comparisons of bleeding events across different antithrombotic treatments after mitral valve repair. A lower risk of bleeding is associated with OAC relative to SAPT

**Fig. 3:**
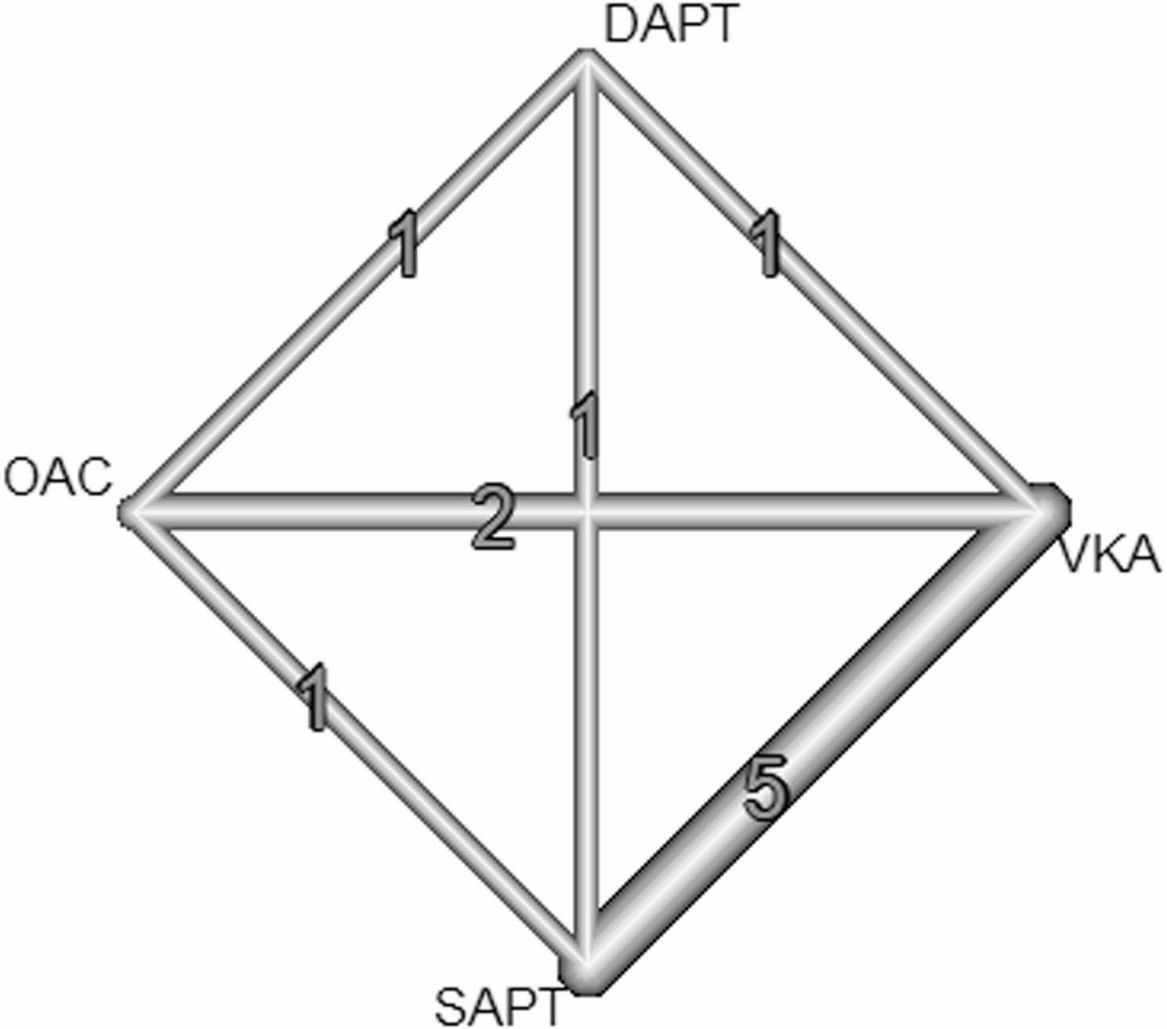
Network Meta-Analysis of Bleeding Events: illustrating the risk ratio comparisons of bleeding events across different antithrombotic treatments after mitral valve repair. A lower risk of bleeding is associated with OAC relative to SAPT

Our network meta-analysis showed that the most effective intervention in decreasing the risk of bleeding after mitral valve repair (both surgical and transcatheter) was OAC in comparison to SAPT (RR = 0.31; 95% CI: [0.11–0.87]). The pooled studies showed moderate heterogeneity (I^2^ = 57.5%, *P* = 0.038). The other interventions showed insignificant results in comparison to SAPT (VKA: RR = 0.79; 95% CI: [0.48; 1.30]), DAPT: (RR = 1.24; 95% CI: [0.51; 3.01]) (Figs. 2 and 3).

**Fig. 4:**
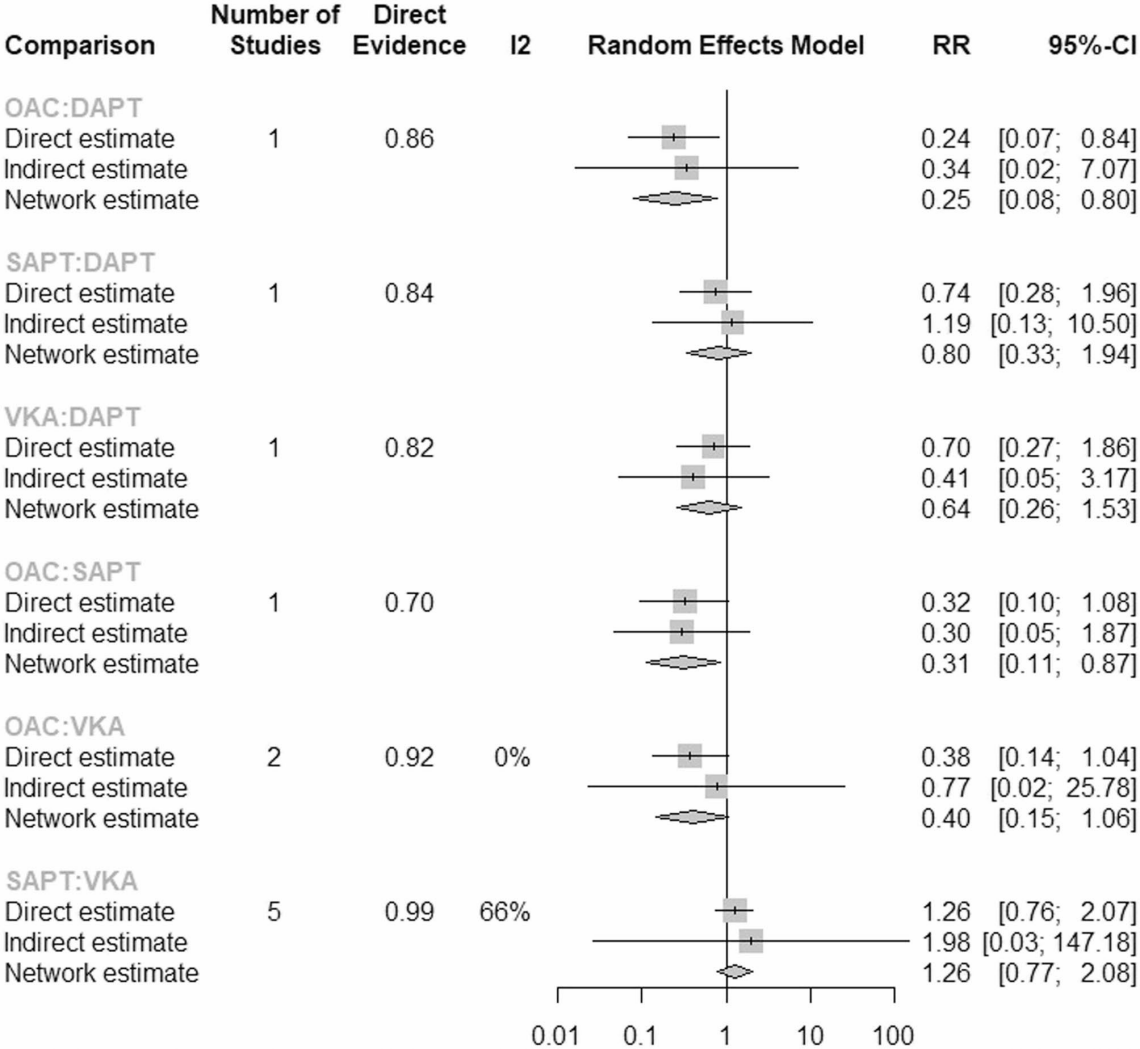
Comparison of Bleeding Risk Between OAC and DAPT: displaying the relative risk of bleeding in patients treated with OAC compared to DAPT, showing a statistically significant reduction in bleeding risk with OAC

Also, OAC showed a lower risk of bleeding in comparison to DAPT (RR = 0.25, 95% CI: [0.08; 0.80] (Fig. 4).

**Fig. 5:**
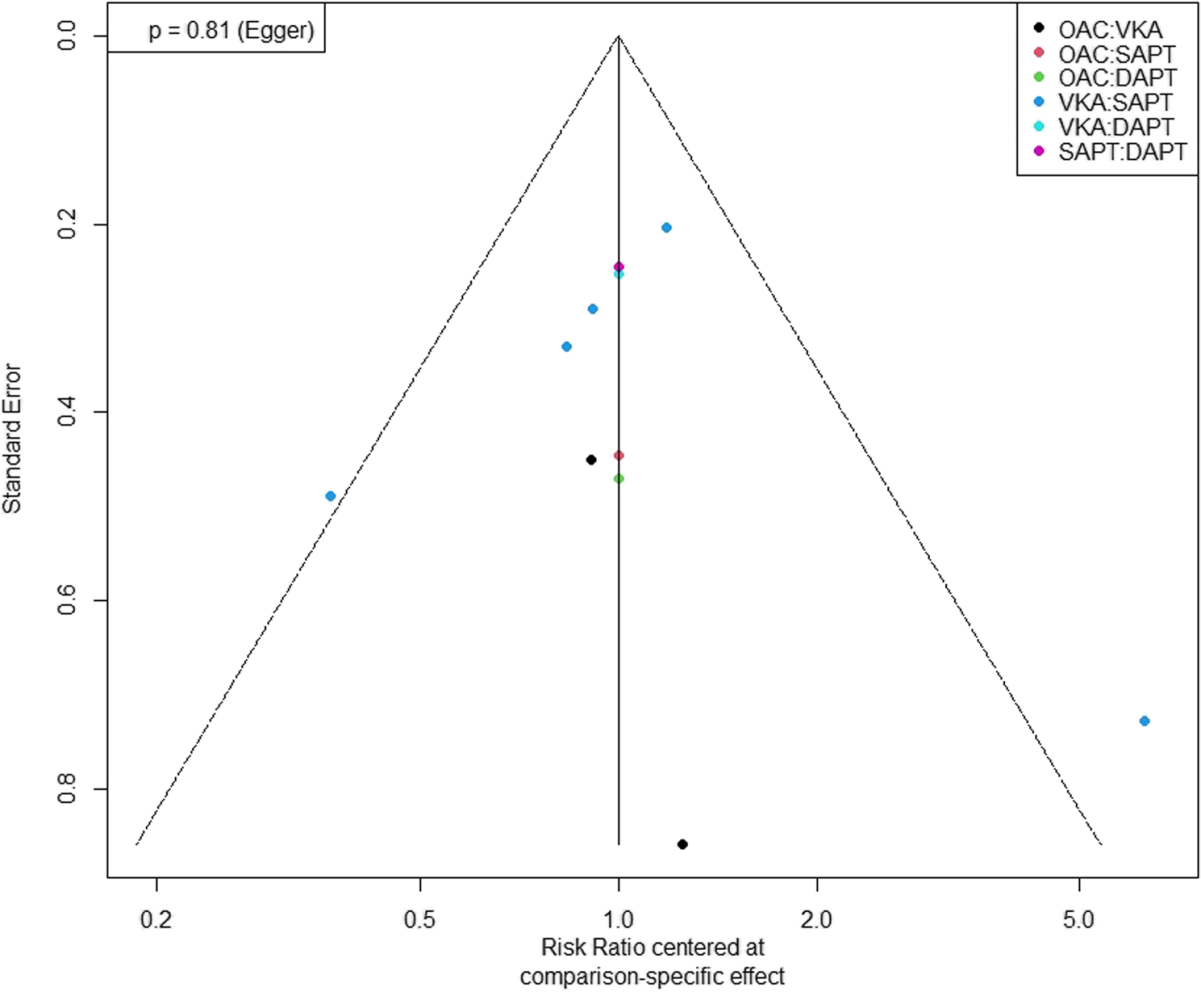
Egger’s Test for Publication Bias: presenting the results of Egger’s test, evaluating potential publication bias across studies included in the meta-analysis of bleeding events

In terms of publication bias, our analysis showed insignificant publication bias (*P*-value of Egger test = 0.81) (Fig. 5).

#### Thromboembolic events

**Fig. 6:**
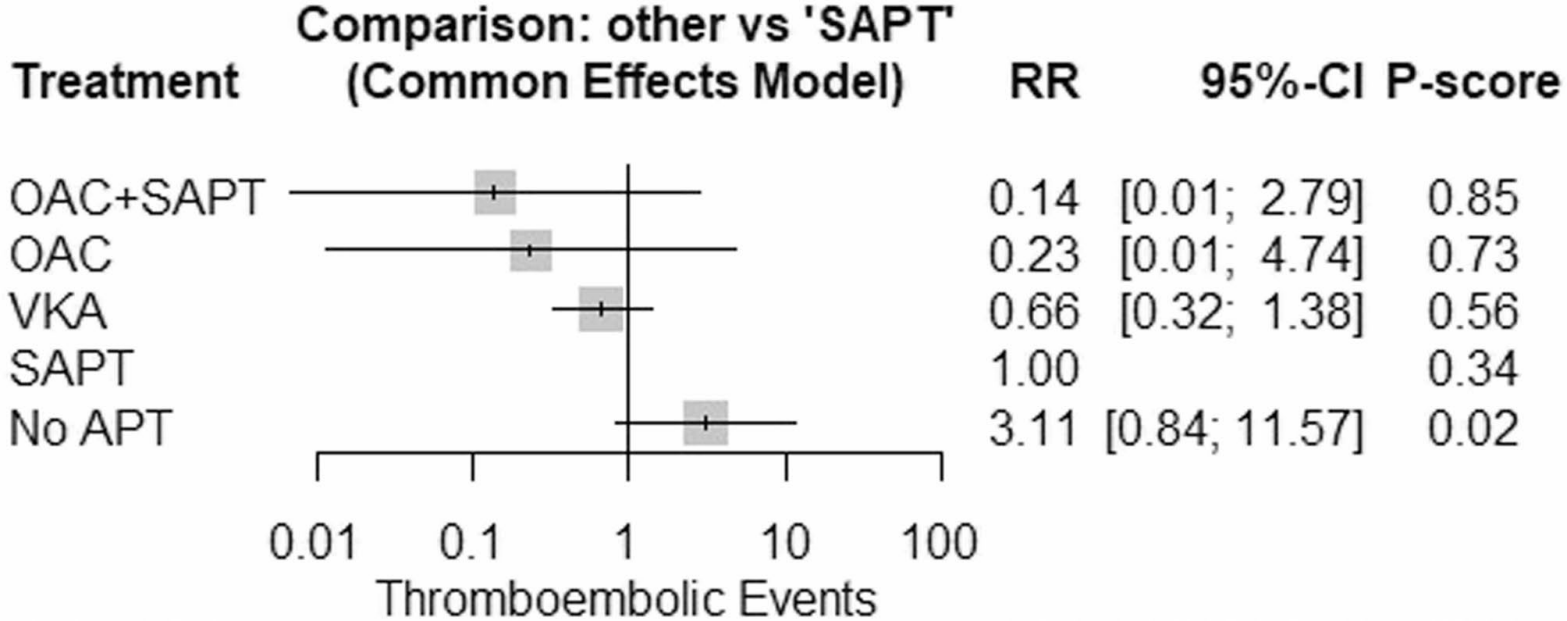
Thromboembolic Events Across Interventions: compareing thromboembolic event rates among various antithrombotic therapies, showing no significant differences in risk

**Fig. 7:**
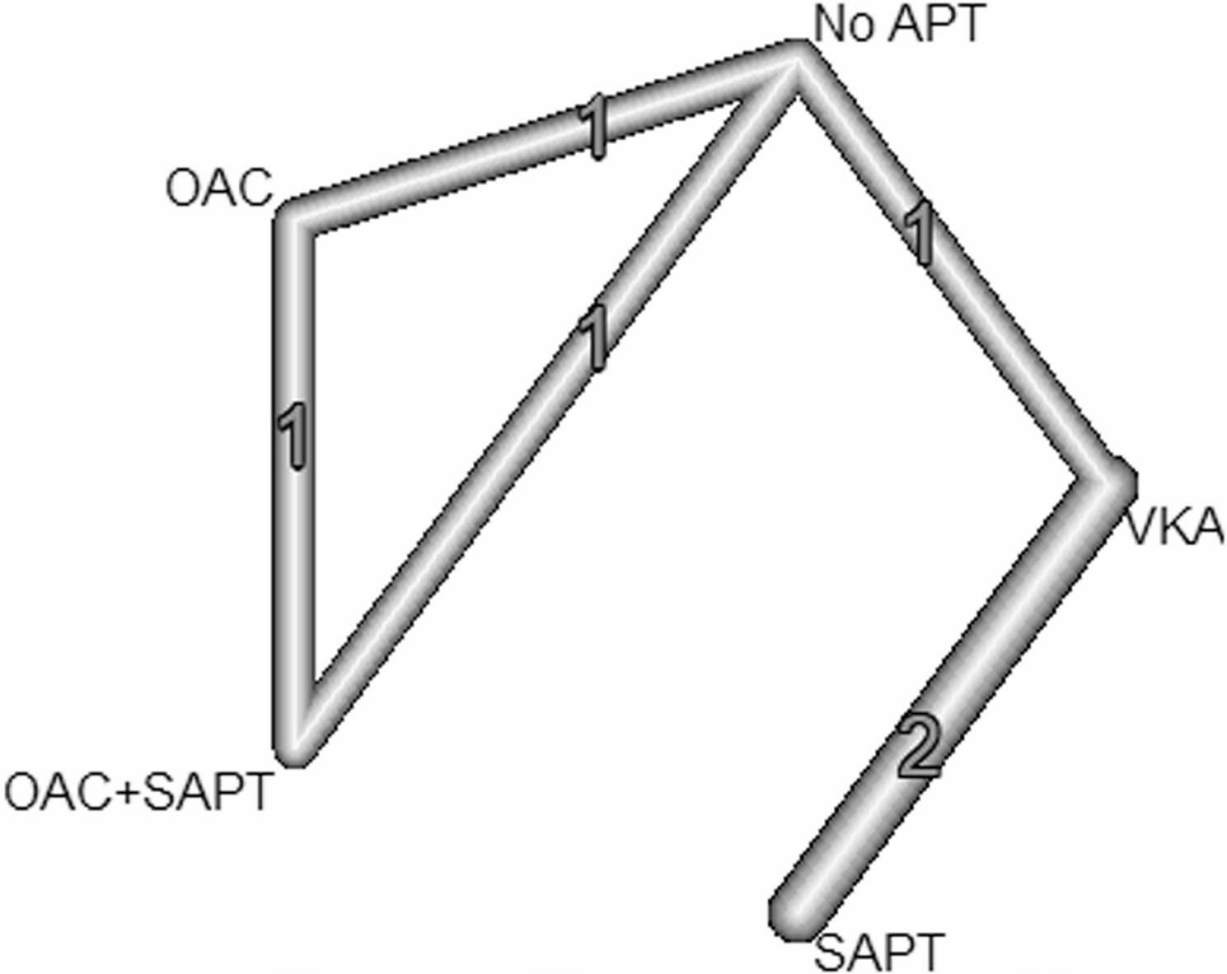
Thromboembolic Events Across Interventions: compareing thromboembolic event rates among various antithrombotic therapies, showing no significant differences in risk

The network meta-analysis showed insignificant results in comparison to (SAPT [OAC + SAPT: RR = 0.14, 95% CI: [0.01; 2.79]), (OAC: RR = 0.23, 95% CI 0.01; 4.74), VKA: RR = 0.66, 95% CI 0.23; 1.38), (No APT: RR = 3.11, 95% CI 0.84; 11.75)] (Figs. 6 and 7).

**Fig. 8:**
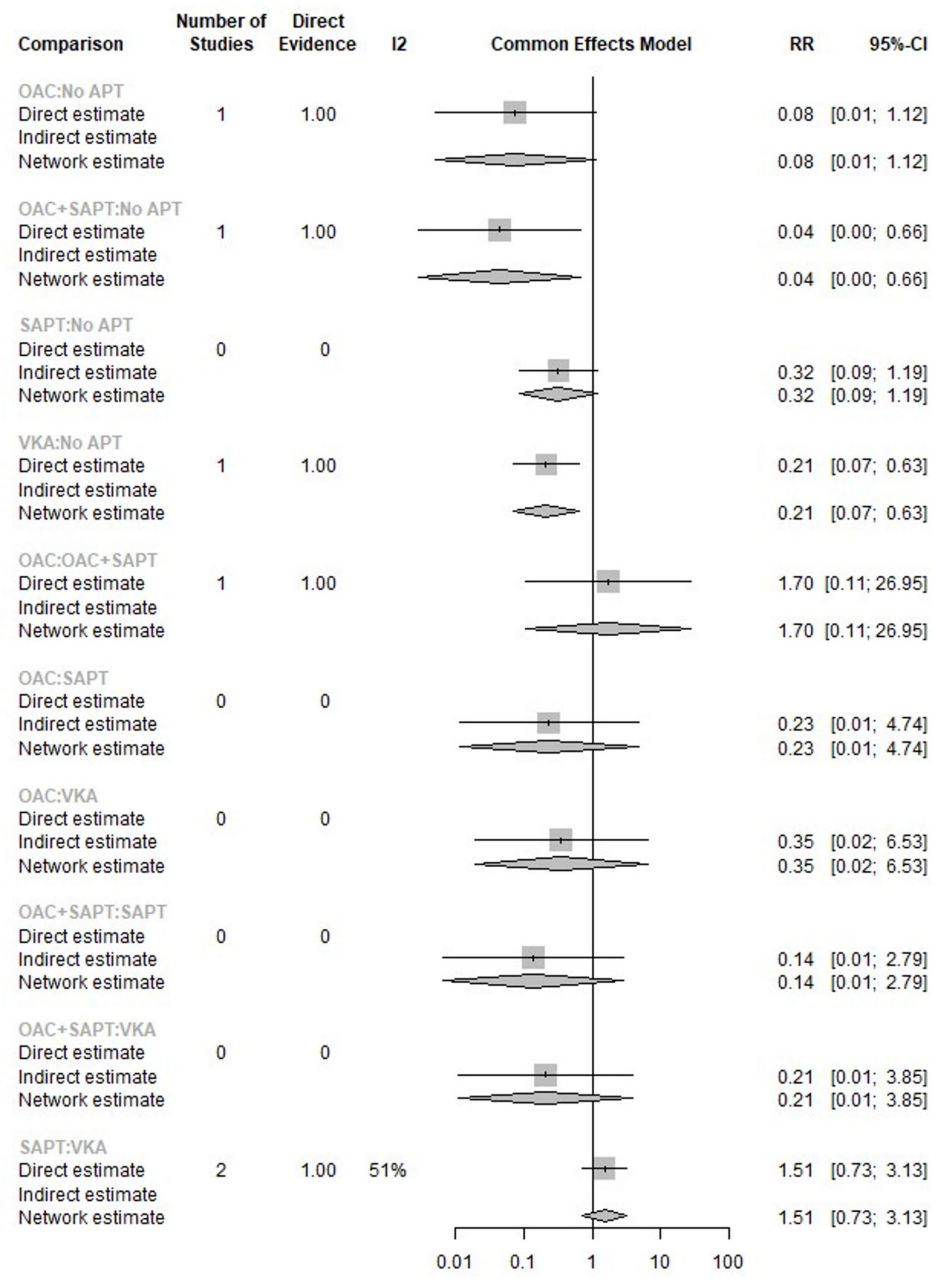
Network meta-analysis comparing the relative risk (RR) of different antithrombotic and anticoagulant strategies.

The head-to-head comparison of these interventions showed insignificant results. The analysis showed no significant heterogeneity (I^2^ = 50.6%, *p* = 0.1546) (Fig. 8).

#### Transient ischemic attacks

**Fig. 9:**
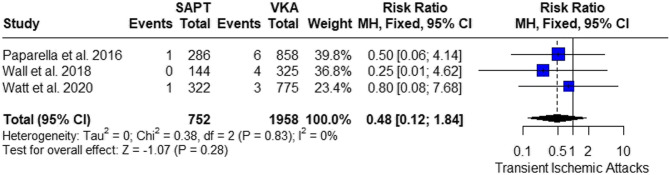
Risk of Transient Ischemic Attacks (TIA): showing the comparative risk of TIA between SAPT and VKA, with no significant difference observed

The meta-analysis showed insignificant results between SAPT and VKA (RR = 0.48, 95% CI 0.12; 1.84, *P* = 0.28). The pooled studies were homogenous (I^2^ = 0%, *p* = 0.83) (Fig. 9).

#### Stroke

**Fig. 10:**
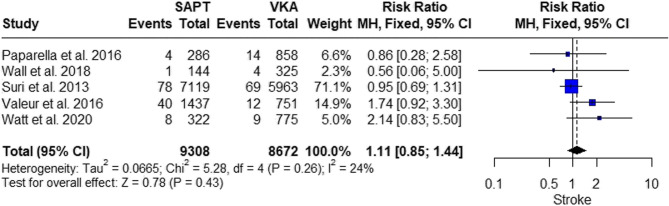
Stroke Risk Comparison: providing a visual comparison of stroke risk between SAPT and VKA post-mitral valve repair

The pooled studies showed insignificant results between SAPT and VKA (RR = 1.11, 95% CI 0.85; 1.44, *P* = 0.43). The pooled studies showed insignificant heterogeneity (I^2^ = 24%, *p* = 0.26) (Fig. 10).

#### 6-month mortality

**Fig. 11:**
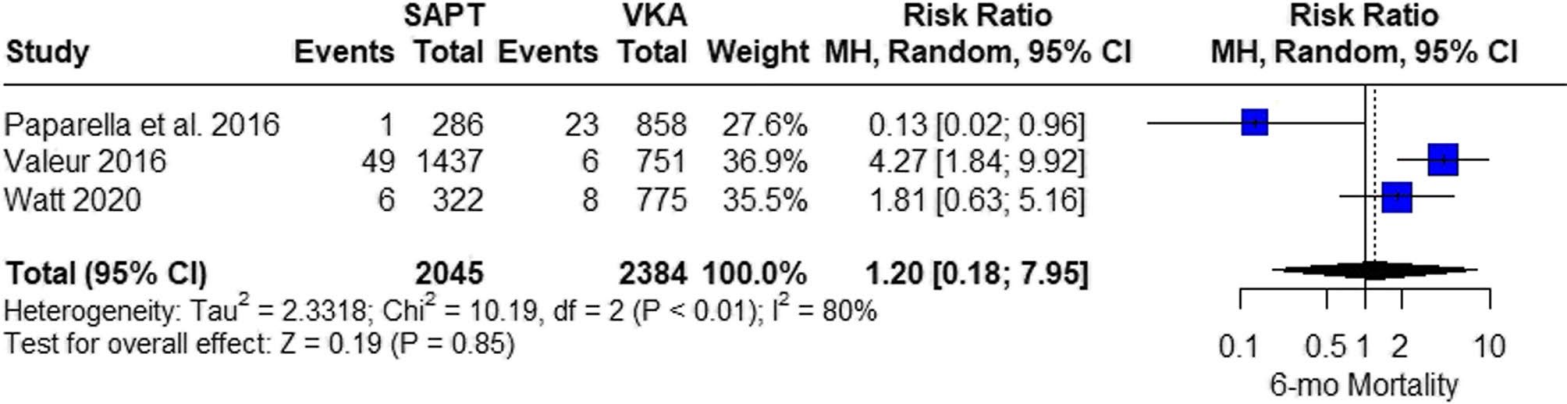
Six-Month Mortality Risk Analysis: comparing six-month mortality risk across different treatments, with a significant finding upon exclusion of one study to reduce heterogeneity

**Fig. 12:**
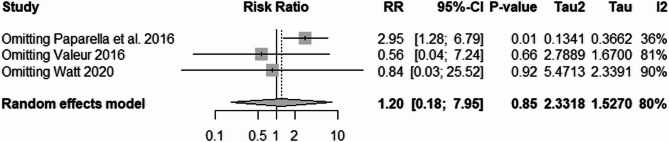
Six-Month Mortality Risk Analysis: comparing six-month mortality risk across different treatments, with a significant finding upon exclusion of one study to reduce heterogeneity

The pooled studies showed insignificant results between SAPT and VKA (RR = 1.20, 95% CI 0.18; 7.95, *P* = 0.85). The pooled studies were heterogeneous (I^2^ = 80%, *p* < 0.01) (Fig. 11). The leave-one-out test was done. The heterogeneity resolved after removing Paparella et al. 2016 [23] and the SAPT showed higher risk of 6-month mortality than VKA (RR = 2.95, 95% CI 1.28; 6.79, I^2^ = 36%) (Fig. 12).

#### Qualitative synthesis for transcatheter MVR

Benito-González et al. 2018 [28] conducted a prospective cohort study on 80 patients who underwent percutaneous MVR with advanced functional class NYHA III-IV, 17.44 months as median follow-up time, and 96.3% procedural success rate. The bleeding events for our included interventions that reported in this study were as follows: DAPT 3/21, VKA 7/21, SAPT 2/21, OAC 3/21. Patients with AF were at high risk of thromboembolic events with HAS-BLED score (mean ± SD: 2.4 ± 1.2) and 40.4% were at high risk of bleeding events. Hohmann et al. 2022 [27] reported MACE, all-cause mortality, combined bleeding endpoint that included intracranial, extra cranial, and gastrointestinal bleeding for 1342 patients with Mean Charlson Comorbidity Index 5.1 for patients who underwent percutaneous MVR using MitraClip. Events of MACE in DAPT, OAC, and No APT were less than 5, 8 and less than 5; respectively. Events of all-cause mortality in DAPT, OAC, and No APT were 31, 37, and 57; respectively. The gastrointestinal bleeding in DAPT, OAC, and No APT was 12, 6, and less than 5; respectively. Körber et al. 2018 [26] reported that percutaneous MVR achieved 94.3% procedural success from total patients 327. They reported bleeding complications according to the definitions published by Mitral Valve Academic Research Consortium (MVARC). Bleeding events in VKA group were 31 from 104 patients, in OAC group were 5 from 49 patients, in SAPT group were 36 from 114 patients and in DAPT group 14 from 33 patients. Seeger et al. 2019 [22] reported no bleeding events in either Apixaban plus aspirin or APT. However, Waechter et al. 2022 [19] reported 16 (2.6%) Bleeding events and 3 (0.5%) thromboembolic events from 609 total cohort who underwent percutaneous MVR.

## Discussion

This systematic review aimed to compare different AT medications after MVR. Including nine studies in this network analysis, we found that all analyzed AT medications were comparable regarding TE events. However, VKA and OAC + SAPT were linked to a reduced incidence of TE events compared to those who received no AT medications. Our network meta-analysis showed that only OAC was associated with a lower risk of bleeding; it showed significant results compared to SAPT and DAPT. Also, there was no significant difference between SAPT and VKA in the risk of TIA, stroke, or six-month mortality.

### Thromboembolic events

Our findings on TE events following MVR suggest that AT medications (VKA and OAC + SAPT) are linked to a reduced incidence of TE events compared to those who did not receive AT medications. However, our study did not find any significant differences between different types of AT medications. These results are consistent with the findings of multiple previous studies.

A multicenter cohort study conducted by Waechter et al. [29] on 609 patients undergoing TMVR using transcatheter edge-to-edge repair (TEER) in Germany found that TE events were similar in patients treated with SAPT, DAPT OAC, OAC + SAPT, and OAC + DAPT (*p* = 1). Other studies conducted in France and the Netherlands have also shown that different AT medications do not differ significantly regarding the incidence of TE. A study conducted by Meurin et al. [30] on 350 patients treated with VKA, ASA, or ASA + VKA after SMVR found that only the absence of AT therapy was associated with an increased risk of TE events in the first six weeks after surgery (hazard ratio (HR) = 6.7, 95% CI: [2.1–21], *p* = 0.0002), while all AT medications were comparable. Similarly, a study by Wall et al. [31] on 469 patients who received VKA or ASA after SMVR found no significant differences in TE rates (three months adjusted HR = 0.82, 95%CI: [0.16, 4.2]).

Although there was no significant difference in TE complications between the different AT medications, the study found that the incidence of TE was less than half in the VKA and OAC + SAPT groups. This suggests that these two AT regimens may provide protection against TE. However, larger studies are necessary to fully evaluate this matter due to the low event rate.

In a retrospective study conducted by Watt et al. [32] on 1097 patients who underwent SMVR, it was found that there was no significant difference in TE between patients treated with VKA and those who did not receive AT treatment (*p* = 0.1). However, excluding patients with AF in Watt et al.‘s study [32] may have led to controversial results. AT medications play a major role in such a population, and even with that exclusion, the TE events were half in the patients who received VKA compared to those who did not (1.5% vs. 3.1%).

Also, our analysis resulted in insignificant results between VKA and SAPT regarding TIA and stroke, which align with that of Paparella et al. 2016 [33], Wall et al. 2018 [31], Watt et al. 2020 [32], Valeur et al. 2016 [34], and Suri et al. 2013 [9].

### Bleeding

Our network meta-analysis showed that the least risk of bleeding was associated with OAC; it showed significant results compared to SAPT and DAPT. Our findings align with that of Körber et al. 2018 [35]. They retrospectively assessed a cohort of 353 patients who underwent PMVR in Germany between November 2012 and June 2016. The least risk of bleeding was associated with OAC (1.6%) ompared to VKA (10.2%), SAPT (11.7%), and DAPT (4.56%). Noohi et al. [36] reported equal bleeding events after OAC (rivaroxaban) (1.9%) and VKA (4%) (*p* = 0.4) in their cohort of patients undergoing SMVR.

Notably, several outcomes in our analysis demonstrated moderate to substantial heterogeneity, particularly in the assessment of bleeding and six-month mortality. This heterogeneity may be attributed to several factors, including variability in patient populations, especially between those undergoing surgical versus transcatheter mitral valve repair. Patients selected for transcatheter procedures often represent a higher surgical risk and may have different comorbidity profiles, which can influence both thrombotic and bleeding risks. Additionally, differences in antithrombotic protocols across studies, such as drug combinations, dosages, and timing of administration, contribute to inter-study variability. Variation in follow-up duration and methodological quality, including retrospective versus prospective design and outcome definitions, may also have played a role.

To explore the source of heterogeneity in the six-month mortality analysis, we conducted a leave-one-out sensitivity analysis, which revealed that the study by Paparella et al. 2016 had a significant impact on both heterogeneity and pooled effect estimates. Upon exclusion of this study, heterogeneity dropped from I² = 80–36%, and a statistically significant increase in mortality risk associated with SAPT compared to VKA emerged (RR = 2.95, 95% CI: 1.28–6.79). This suggests that Paparella et al. may have introduced heterogeneity due to unique patient characteristics, treatment practices, or reporting methodologies. These findings emphasize the importance of sensitivity analyses in interpreting meta-analytic data and suggest that conclusions regarding mortality should be interpreted cautiously, especially when influenced by outlier studies.

Watt et al. 2020 reported that VKA was associated with a significant decrease in the risk of bleeding than the SAPT (0.9% vs. 3.1%, *p* = 0.007). One possible explanation for this could be that patients who were deemed more susceptible to bleeding incidents after surgery were less likely to be prescribed anticoagulants based on the surgeon’s preference. Additionally, a significant proportion of patients in the non-anticoagulated group experienced pericardial tamponade bleeding (*p* = 0.045), which further supports the previous explanation when compared to the VKA group.

Currently, a lack of recent data clarifies this issue with the use of OAC. However, we can assume that OACs have a lower risk of major bleeding than VKA based on the results of landmark studies that compared them to valvular heart disease patients with valvular heart diseases and prosthetic valves [37, 38].

Given the ongoing debate on postoperative anticoagulation and the variability in clinical practices, this study underscores the significance of further exploring this matter through RCTs. Our network meta-analysis has major limitations due to the heterogeneity between the studies that were pooled. There are several reasons for this. Firstly, there is a significant difference between the demographic profiles of PMVR and SMVR candidates. Currently, PMVR is recommended for patients with a higher surgical risk. As we had limited data from studies using PMVR, we were unable to address this issue through subgroup analysis. Secondly, the empirical AT treatment of MVR patients is highly variable in real-world settings because of the lack of evidence-based data available. It is directly associated with the institution and surgeon’s experience and choice. Thirdly, the included studies were retrospective in design, which makes them more susceptible to confounding factors such as surgeon experience. Finally, a third of the studies we included had a high risk of bias.

AT regimens of OAC may be highly relevant for patients with AF. However, it remains unclear whether OAC will have the same properties when used for patients with less prevalent AF, such as SMVR candidates. We were not able to study this subgroup due to insufficient data.

Our analysis demonstrated that AT medications had beneficial effects, but RCTs should be conducted to account for confounding factors in observational studies and determine which group of MVR candidates would benefit from AT medications. Although the limited number of studies under each treatment group and the high level of heterogeneity between patients mean that our results should be taken as hypothesis-formulating, AT medications did not increase the risk for adverse events and may be beneficial. Additionally, due to the limited number of high-quality studies available for each type of mitral valve repair individually, this approach allowed us to include a broader range of evidence and enhance the statistical power; however, we acknowledge the inherent differences between these procedural modalities as patient risk profiles and perioperative management.

We recognize this as a potential limitation and have emphasized the need for future studies that separately analyze antithrombotic strategies in more homogenous populations undergoing either surgical or transcatheter repair. In addition, While we used the NOS to assess the quality of included observational studies due to its broad acceptance and simplicity, we acknowledge that prospective protocol registration and the ROBINS-I tool offer a more detailed risk-of-bias evaluation tailored to non-randomized intervention studies. Future systematic reviews might benefit from applying both tools, particularly when assessing the causal impact of interventions. Our choice of NOS was aimed at consistency with previous evidence in similar settings and for better comparability of findings.

### Conclusion

Our analysis indicates that OACs are associated with a lower risk of bleeding compared to single or dual antiplatelet therapy after mitral valve repair, while VKAs and OAC + SAPT may offer better protection against thromboembolic events than no therapy. However, differences between antiplatelet and anticoagulant strategies were not significant for stroke, TIA, or short-term mortality in most comparisons. These findings highlight the need for individualized decision-making, particularly in patients with atrial fibrillation or elevated thromboembolic risk. Current evidence remains limited and inconsistent, underscoring the need for RCTs to guide antithrombotic therapy following mitral valve repair.

## Supplementary Information

Supplementary Material 1.

## Data Availability

No datasets were generated or analysed during the current study.

## Abbreviations

MR: Mitral Regurgitation
MV: Mitral Valve
SMVR: Surgical Mitral Valve Repair
PMVR: Percutaneous Mitral Valve Repair
TMVR: Transcatheter Mitral Valve Repair
TE: Thromboembolic
AT: Antithrombotic
ESC: European Society of Cardiology
EACTS: European Association for Cardio-Thoracic Surgery
ACC: American College of Cardiology
AHA: American Heart Association
AF: Atrial Fibrillation
RCT: Randomized Controlled Trial
NOS: Newcastle-Ottawa Scale
ASA: Acetylsalicylic Acid (Aspirin)
VKA: Vitamin K Antagonist
OAC: Oral Anticoagulant
SAPT: Single Antiplatelet Therapy
DAPT: Dual Antiplatelet Therapy
No APT: No Antiplatelet Therapy
TIA: Transient Ischemic Attack
CI: Confidence Interval
RR: Risk Ratio
PRISMA: Preferred Reporting Items for Systematic Reviews and Meta-Analyses
MVARC: Mitral Valve Academic Research Consortium
MACE: Major Adverse Cardiac Events
NYHA: New York Heart Association
SD: Standard Deviation
HR: Hazard Ratio
